# Supplementary material for: Large-scale integration of single-cell transcriptomic data captures transitional progenitor states in mouse skeletal muscle regeneration
Source: Commun Biol. 2021 Nov 12;4:1280. doi: 10.1038/s42003-021-02810-x (PMC8589952; doi:10.1038/s42003-021-02810-x)
Supplement: Supplementary file 2 — Supplementary Information [file 42003_2021_2810_MOESM2_ESM.pdf]

Supplementary Information for

Large-scale integration of single-cell transcriptomic data captures transitional progenitor states in mouse skeletal muscle regeneration

David W. McKellar[1], Lauren D. Walter[2], Leo T. Song[1], Madhav Mantri[3], Michael F.Z. Wang[3], Iwijn De Vlaminck[1,#,%], and Benjamin D. Cosgrove[1,#,%]

[1]Meinig School of Biomedical Engineering, Cornell University, Ithaca, NY 14853, USA
[2]Department of Molecular Biology & Genetics, Cornell University, Ithaca, NY 14853, USA
[3]Department of Computational Biology, Cornell University, Ithaca, NY 14853, USA

[#] These authors contributed equally
[%] Correspondence to I.D. (id93@cornell.edu) and B.D.C. (bdc68@cornell.edu)

Table S1. Metadata for datasets included in this study.

Citation	GEO Accession	Age (mo)	Injury (dpi)	Muscle(s)	Cell Isolation Procedures	10x Chemistry	# Samples	# Cells (After QC)
De Micheli et al, *Cell Reports*, 2020	GSE143437, GSE143435	4-7	Notexin (0, 2, 5, 7)	Tibialis anterior	Dissociated Whole Muscle	v2 & v3	14	65,234
De Micheli et al (*)	GSE159500	7	Notexin (2 & 7)	Tibialis anterior	Dissociated Whole Muscle	v3	2	7,027
McKellar, Walter et al (*)	GSE162172	20	Notexin (0, 1, 2, 3.5, 5, 7)	Tibialis anterior	Dissociated Whole Muscle	v3	21	89,382
Dell'Orso et al, *Development*, 2019	GSE126834	3	Notexin (0, 2.5)	Tibialis anterior	Dissociated Whole Muscle & FACS	v2	6	7,582
Giordani et al, *Molec Cell*, 2019	GSE110878	2	-	Hindlimb Muscles	Dissociated Whole Muscle	v2	2	11,375
Tabula Muris, *Nature*, 2018	GSE109774	3	-	Tibialis anterior	Dissociated Whole Muscle	v2	2	4,739
Tabula Muris Senis, *Nature*, 2020	GSE149590	18-24	-	Tibialis anterior	Dissociated Whole Muscle	v2	10	24,050
Li et al, *The EMBO Journal*, 2019	GSE134540	3	-	Tibialis anterior & Soleus	FACS (MuSCs)	v2	3	9,414
Jin et al, *JCI Insights*, 2018	GSE113111	3	Cardiotoxin (4)	Hindlimb Muscles	FACS (Macrophages)	v2	2	7,631
Rubenstein et al, *Scientific Reports*, 2020	GSE138707	3.5	-	Quadriceps/ Diaphragm	Dissociated Whole Muscle	v3	2	6,841
Kimmel et al, *Development*, 2020	GSE143476	3 & 18	-	Hindlimb Muscles	FACS (MuSCs)	v2	16	21,524
Verma et al, *bioRxiv*, 2021	GSE129057	2	Cardiotoxin (0, 3)	Hindlimb Muscles	FACS (Endo. & MuSCs)	v2	2	1,667
Stepien et al, *J Immunol*, 2020	GSE144270	2	Cardiotoxin (0, 3)	Tibialis anterior	Dissociated Whole Muscle	v2	5	9,793
Oprescu et al, *iScience*, 2020	GSE138826	3	Cardiotoxin (0, 0.5, 2, 3.5, 5, 7, 10, 21)	Tibialis anterior	FACS Whole Muscle	v3	7	47,813
Kalucka et al, *Cell*, 2020	**	2	-	EDL + Soleus	FACS (Endothelial)	v2	2	3,486
Petrany et al, *Nat Comm*, 2020	GSE147127	10d-30	-	TA + Soleus	Nuclei (Whole Muscle)	v3	6	30,380
Dos Santos et al, *Nat Comm*, 2020	GSE150065	2	-	TA, Soleus, & Quadriceps	Nuclei (Whole Muscle)	v3	4	2,075
Wang et al, *PNAS*, 2020	GSE142480	2.5	-	Quadriceps/ Diaphragm	FACS (Macrophages)	v2	2	6,351
Chemello et al, *PNAS*, 2020	GSE156498	1	-	Tibialis anterior	Nuclei (Whole Muscle)	v3.1	1	3,251
Yartseva et al, *Cell Rep*, 2020	GSE142581	3.5	Cardiotoxin (0, 4)	Tibialis anterior	FACS (MuSCs)	v2	2	5,396
						Total:	**111**	**365,011**

* New data first reported in this study.
** Available at ArrayExpress, accession number E-MTAB-8077

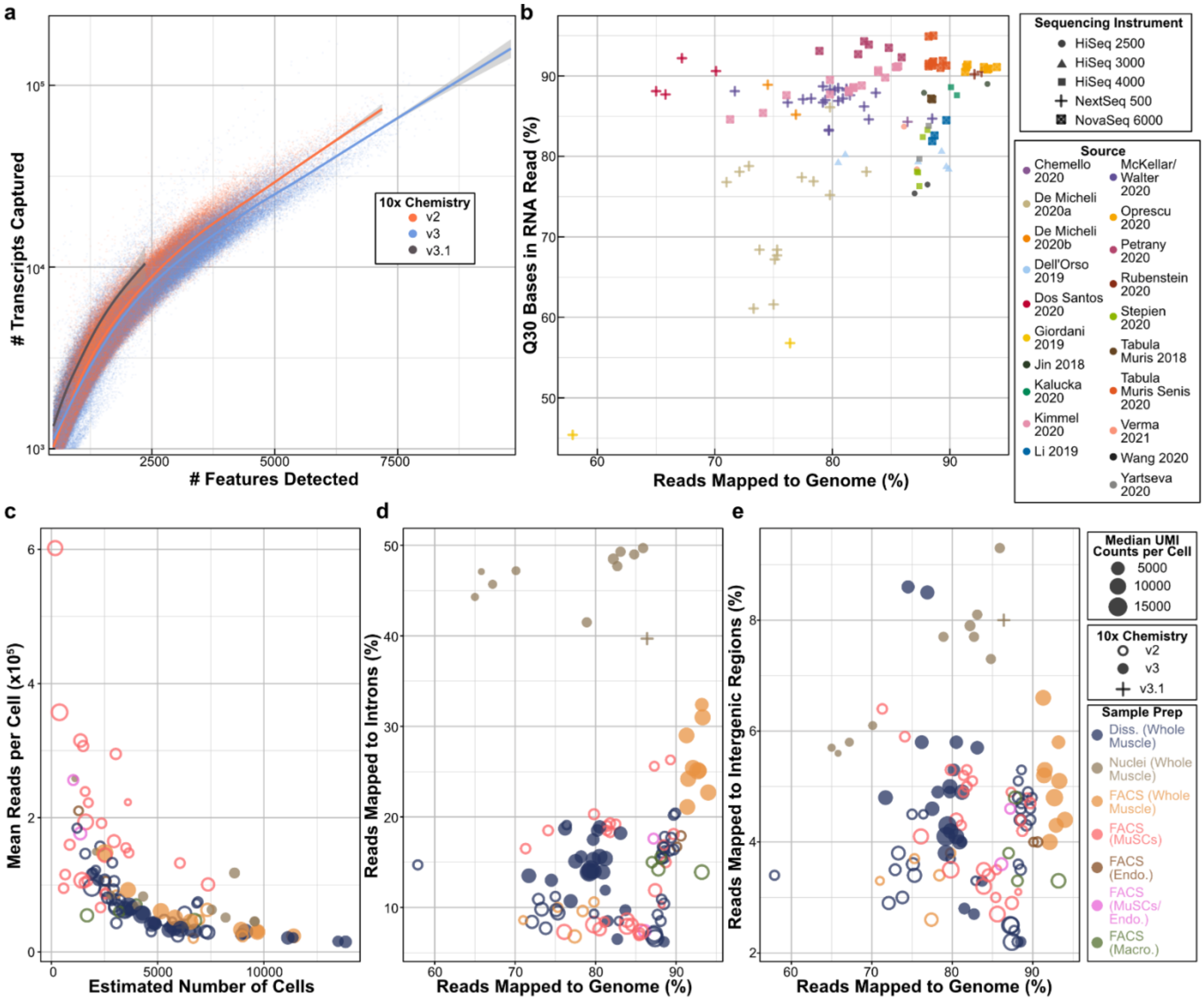

Figure S1. Sequencing metrics and sample material drive batch effects in single-cell RNA sequencing data. (a) The number of features and transcripts detected in each droplet, after quality filtering, is similar between versions 2 and 3 of the 10x Chromium chemistry. **(b)** Quality of sequencing, as determined by mappability of the library and Q30 bases inside the RNA portion of the read, may also drive batch effects. **(c)** Sequencing depth is often sacrificed in favor of higher cell counts. Single-nuclei data are enriched with **(d)** nascent transcripts and **(e)** reads outside of annotated genes.

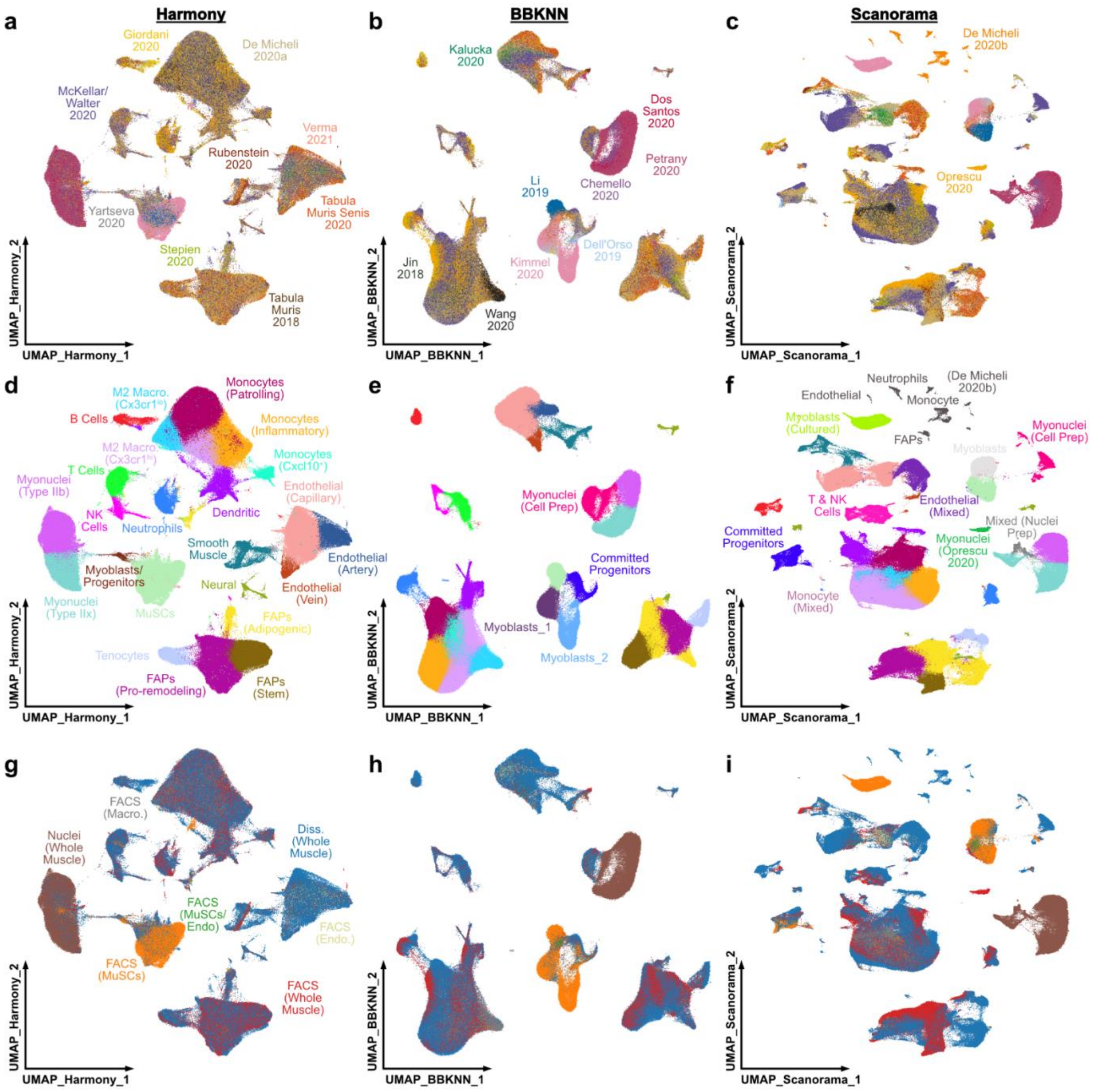

Figure S2. Comparison of batch-correction methods. UMAP plots generated after integration with either Harmony, BBKNN, or Scanorama are shown. Plots are colored and labeled by **(a-c)** data source, **(d-f)** annotated cell type, or **(g-i)** sample preparation strategy. Cell types were annotated independently for each batch-correction method (see **Methods**).

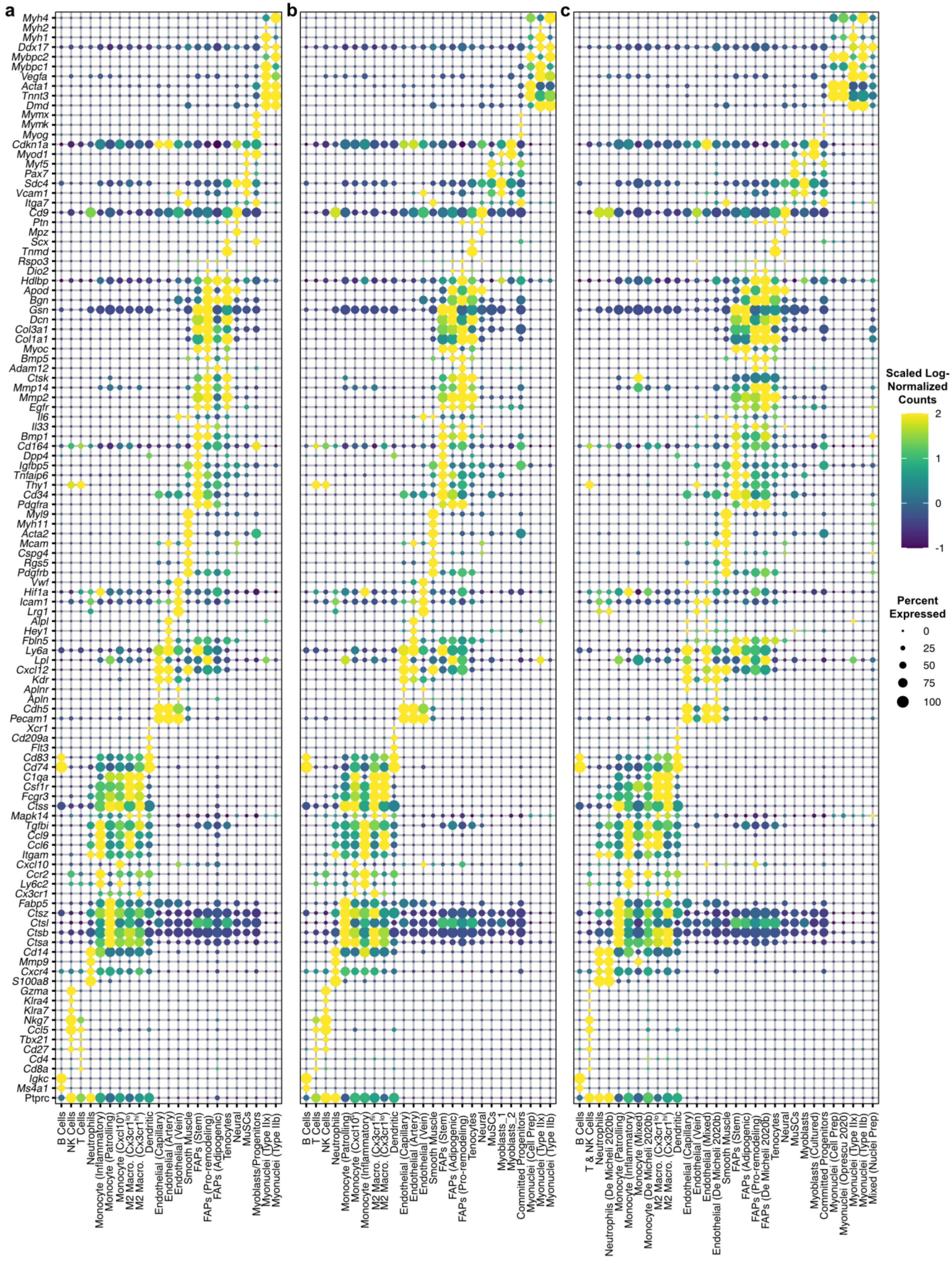

a
b
c
Myh4
Myh2
Myh1
Ddx17
Mybpc2
Mybpc1
Vegfa
Acta1
Tnnt3
Dmd
Mymx
Mymk
Myog
Cdkn1a
Myod1
Myf5
Pax7
Sdc4
Vcam1
Itga7
Cd9
Ptn
Mpz
Scx
Tnmd
Rspo3
Dio2
Hdlbp
Apod
Bgn
Gsn
Dcn
Col3a1
Col1a1
Myoc
Bmp5
Adam12
Ctsk
Mmp14
Mmp2
Egfr
Il6
Il33
Bmp1
Cd164
Dpp4
Igfbp5
Tnfaip6
Thy1
Cd34
Pdgfra
Myl9
Myh11
Acta2
Mcam
Cspg4
Rgs5
Pdgfrb
Vwf
Hif1a
Icam1
Lrg1
Alpl
Hey1
Fbln5
Ly6a
Lpl
Cxcl12
Kdr
Aplnr
Apln
Cdh5
Pecam1
Xcr1
Cd209a
Flt3
Cd83
Cd74
C1qa
Csf1r
Fcgr3
Ctss
Mapk14
Tgfbi
Ccl9
Ccl6
Itgam
Cxcl10
Ccr2
Ly6c2
Cx3cr1
Fabp5
Ctsz
Ctsl
Ctsb
Ctsa
Cd14
Mmp9
Cxcr4
S100a8
Gzma
Klra4
Klra7
Nkg7
Ccl5
Tbx21
Cd27
Cd4
Cd8a
Igkc
Ms4a1
Ptprc
B Cells
NK Cells
T Cells
Neutrophils
Monocyte (Inflammatory)
Monocyte (Patrolling)
Monocyte (Cxcl10+)
M2 Macro. (Cx3cr1lo)
M2 Macro. (Cx3cr1hi)
Dendritic
Endothelial (Capillary)
Endothelial (Artery)
Endothelial (Vein)
Smooth Muscle
FAPs (Stem)
FAPs (Pro-remodeling)
FAPs (Adipogenic)
Tenocytes
Neural
MuSCs
Myoblasts/Progenitors
Myonuclei (Type IIx)
Myonuclei (Type IIb)
B Cells
T Cells
NK Cells
Neutrophils
Monocyte (Patrolling)
Monocyte (Cxcl10+)
Monocyte (Inflammatory)
M2 Macro. (Cx3cr1hi)
M2 Macro. (Cx3cr1lo)
Dendritic
Endothelial (Capillary)
Endothelial (Artery)
Endothelial (Vein)
Smooth Muscle
FAPs (Stem)
FAPs (Adipogenic)
FAPs (Pro-remodeling)
Tenocytes
Neural
MuSCs
Myoblasts_1
Myoblasts_2
Committed Progenitors
Myonuclei (Cell Prep)
Myonuclei (Type IIx)
Myonuclei (Type IIb)
B Cells
T & NK Cells
Neutrophils
Neutrophils (De Micheli 2020b)
Monocyte (Patrolling)
Monocyte (Inflammatory)
Monocyte (Mixed)
Monocyte (De Micheli 2020b)
M2 Macro. (Cx3cr1lo)
M2 Macro. (Cx3cr1hi)
Dendritic
Endothelial (Capillary)
Endothelial (Vein)
Endothelial (Mixed)
Endothelial (De Micheli 2020b)
Smooth Muscle
FAPs (Stem)
FAPs (Adipogenic)
FAPs (Pro-remodeling)
FAPs (De Micheli 2020b)
Tenocytes
Neural
MuSCs
Myoblasts
Myoblasts (Cultured)
Committed Progenitors
Myonuclei (Cell Prep)
Myonuclei (Oprescu 2020)
Myonuclei (Type IIx)
Myonuclei (Type IIb)
Mixed (Nuclei Prep)
Scaled Log-Normalized Counts
2
1
0
-1
Percent Expressed
0
25
50
75
100

Figure S3. Canonical marker gene expression identifies cellular subtypes in clustering results from three batch-correction methods. Dot plots show the expression of 115 genes, curated from a literature search for cell type markers in skeletal muscle. Cell types shown were generated through shared nearest neighbor clustering on the output values from **(a)** Harmony, **(b)** BBKNN, and **(c)** Scanorama batch-correction algorithms. Average gene expression values are scaled (via Seurat).

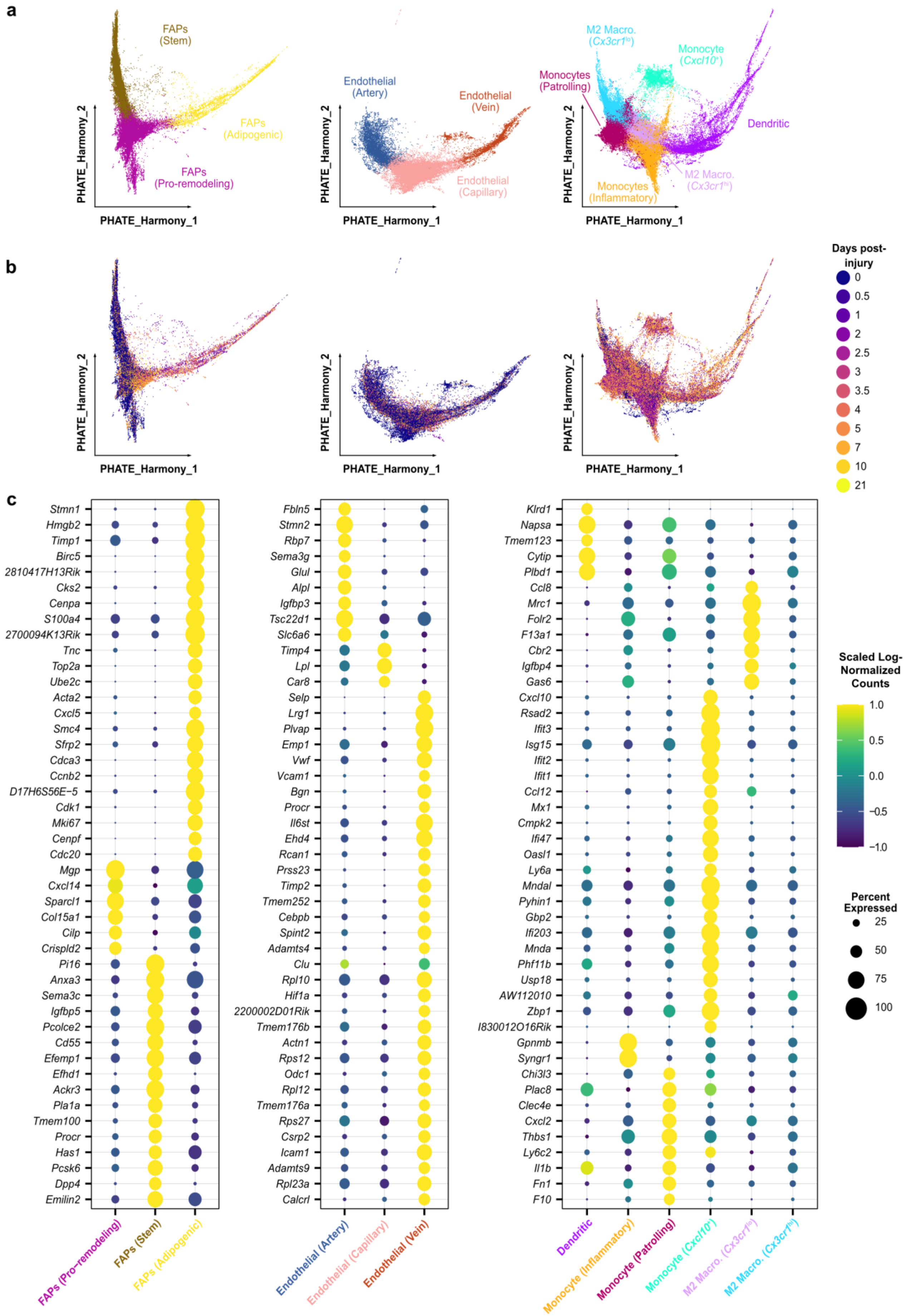

a
FAPs (Stem)
FAPs (Adipogenic)
FAPs (Pro-remodeling)
Endothelial (Artery)
Endothelial (Vein)
Endothelial (Capillary)
M2 Macro. (Cx3cr1hi)
Monocyte (Cxcl10+)
Monocytes (Patrolling)
Dendritic
M2 Macro. (Cx3cr1lo)
Monocytes (Inflammatory)
PHATE_Harmony_2
PHATE_Harmony_1
b
Days post-injury
0
0.5
1
2
2.5
3
3.5
4
5
7
10
21
c
Stmn1
Hmgb2
Timp1
Birc5
2810417H13Rik
Cks2
Cenpa
S100a4
2700094K13Rik
Tnc
Top2a
Ube2c
Acta2
Cxcl5
Smc4
Sfrp2
Cdca3
Ccnb2
D17H6S56E−5
Cdk1
Mki67
Cenpf
Cdc20
Mgp
Cxcl14
Sparcl1
Col15a1
Cilp
Crispld2
Pi16
Anxa3
Sema3c
Igfbp5
Pcolce2
Cd55
Efemp1
Efhd1
Ackr3
Pla1a
Tmem100
Procr
Has1
Pcsk6
Dpp4
Emilin2
FAPs (Pro−remodeling)
FAPs (Stem)
FAPs (Adipogenic)
Fbln5
Stmn2
Rbp7
Sema3g
Glul
Alpl
Igfbp3
Tsc22d1
Slc6a6
Timp4
Lpl
Car8
Selp
Lrg1
Plvap
Emp1
Vwf
Vcam1
Bgn
Procr
Il6st
Ehd4
Rcan1
Prss23
Timp2
Tmem252
Cebpb
Spint2
Adamts4
Clu
Rpl10
Hif1a
2200002D01Rik
Tmem176b
Actn1
Rps12
Odc1
Rpl12
Tmem176a
Rps27
Csrp2
Icam1
Adamts9
Rpl23a
Calcrl
Endothelial (Artery)
Endothelial (Capillary)
Endothelial (Vein)
Klrd1
Napsa
Tmem123
Cytip
Plbd1
Ccl8
Mrc1
Folr2
F13a1
Cbr2
Igfbp4
Gas6
Cxcl10
Rsad2
Ifit3
Isg15
Ifit2
Ifit1
Ccl12
Mx1
Cmpk2
Ifi47
Oasl1
Ly6a
Mndal
Pyhin1
Gbp2
Ifi203
Mnda
Phf11b
Usp18
AW112010
Zbp1
I830012O16Rik
Gpnmb
Syngr1
Chi3l3
Plac8
Clec4e
Cxcl2
Thbs1
Ly6c2
Il1b
Fn1
F10
Dendritic
Monocyte (Inflammatory)
Monocyte (Patrolling)
Monocyte (Cxcl10+)
M2 Macro. (Cx3cr1lo)
M2 Macro. (Cx3cr1hi)
Scaled Log-Normalized Counts
1.0
0.5
0.0
−0.5
−1.0
Percent Expressed
25
50
75
100

Figure S4. Sub-populations trajectories and differentially expressed marker genes. (a) PHATE visualization of FAP, endothelial, and myeloid immune cell sub-populations. Cells are colored by cell type, as shown in **Fig. 1c**. **(b)** PHATE visualization of these cell sub-populations shown by injury time points based on data source. **(c)** Dot plot showing the top 45 differentially expressed marker genes between each cell subtype across all data sources in the compendium. Average gene expression values are scaled (via Seurat). See **Methods** and **Sup. Files 4-6** for additional details.

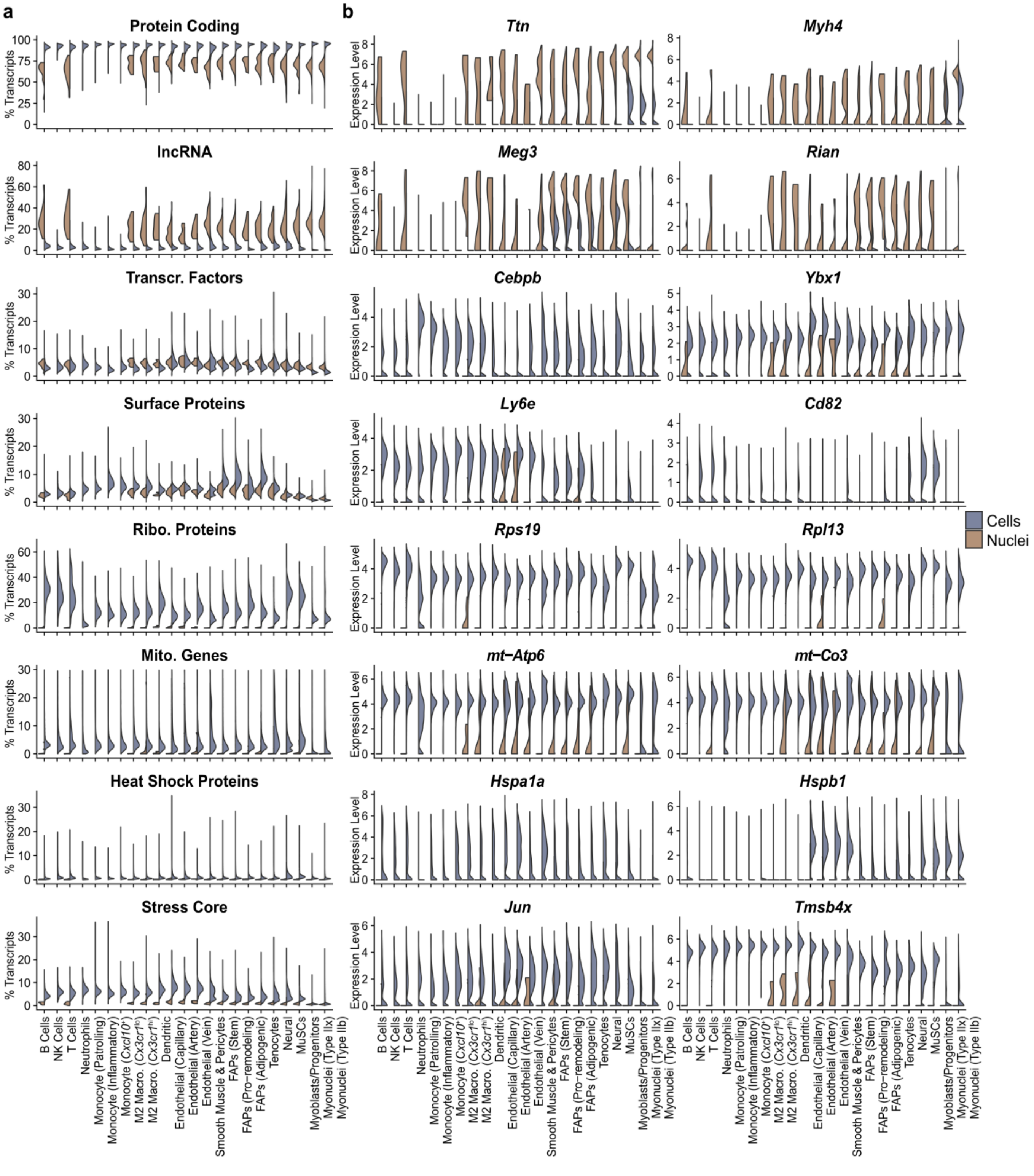

Figure S5. Cell type-specific differences in gene detection between single-cell and single-nucleus RNA sequencing. (a) For each cell type, the differential capture of select gene sets, organized by their encoded protein functions, are shown between cells and nuclei in paired violin plots. The y-axis shows the percent of total transcripts within each single cell/nucleus. **(b)** Example genes from each gene set shown in (a) are reported in the same row.

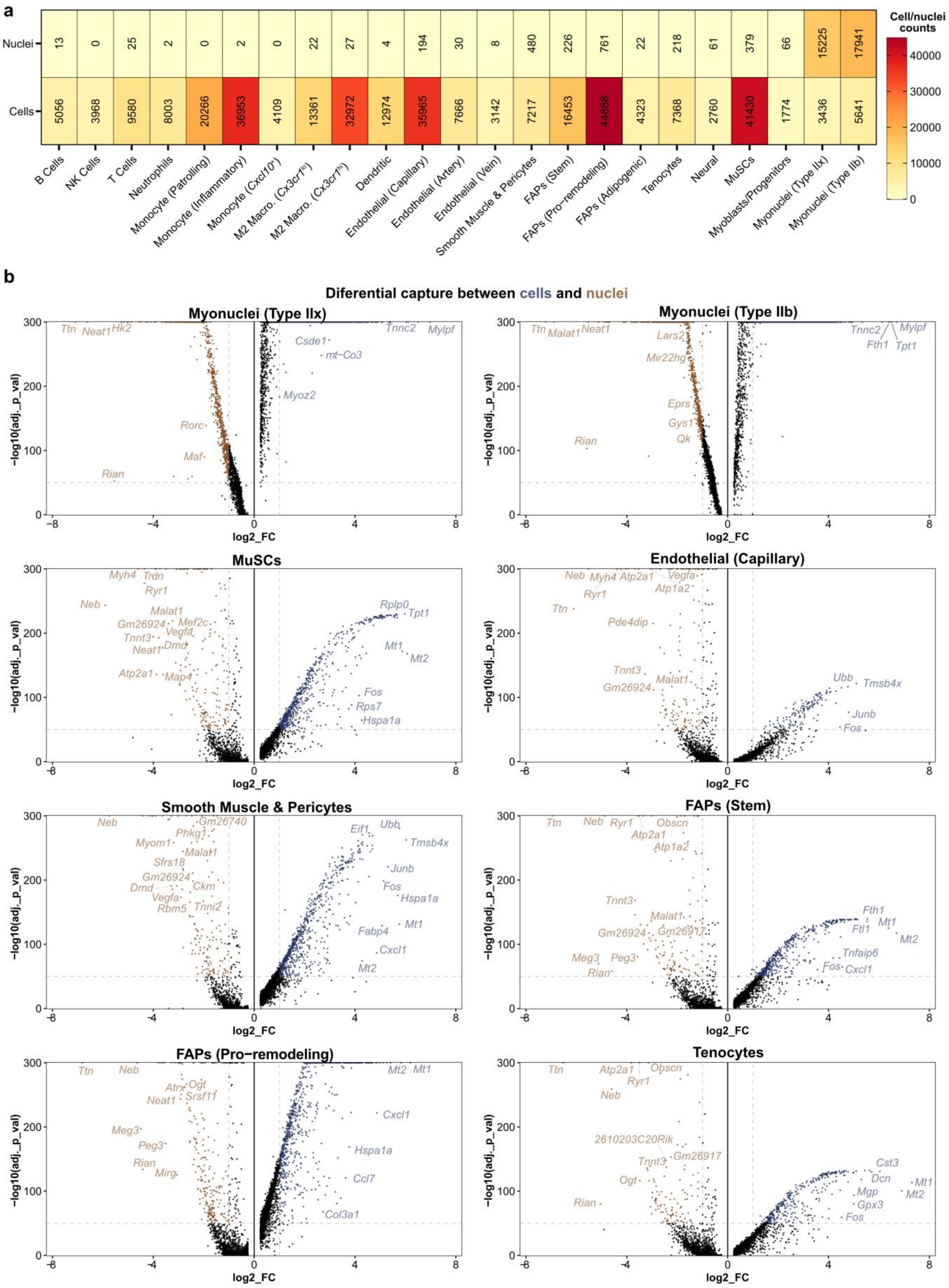
a
Nuclei
Cells
Cell/nuclei counts
B Cells
NK Cells
T Cells
Neutrophils
Monocyte (Patrolling)
Monocyte (Inflammatory)
Monocyte (Cxcl10+)
M2 Macro. (Cx3cr1lo)
M2 Macro. (Cx3cr1hi)
Dendritic
Endothelial (Capillary)
Endothelial (Artery)
Endothelial (Vein)
Smooth Muscle & Pericytes
FAPs (Stem)
FAPs (Pro-remodeling)
FAPs (Adipogenic)
Tenocytes
Neural
MuSCs
Myoblasts/Progenitors
Myonuclei (Type IIx)
Myonuclei (Type IIb)
13 0 25 2 0 2 0 22 27 4 194 30 8 480 226 761 22 218 61 379 66 15225 17941
5056 3968 9580 8003 20266 36953 4109 13361 32972 12974 35965 7666 3142 7217 16453 44888 4323 7368 2760 41430 1774 3436 5641
b
Diferential capture between cells and nuclei
Myonuclei (Type IIx)
Myonuclei (Type IIb)
MuSCs
Endothelial (Capillary)
Smooth Muscle & Pericytes
FAPs (Stem)
FAPs (Pro−remodeling)
Tenocytes
−log10(adj._p_val)
log2_FC

Figure S6. Cell type-specific differential gene detection between single cells and single-nuclei. (a) Abundance of single-cells and single-nuclei by cell type, as determined after Harmony integration, across all data sources in the compendium. **(b)** Differential gene analysis using a using Wilcoxon Rank Sum Test between single-cell and single-nucleus data by cell type for 8 most abundant cell types in both assay sources. Differential expression are results presented as volcano plots with fold-change ratio defined as single-cell over single-nucleus.

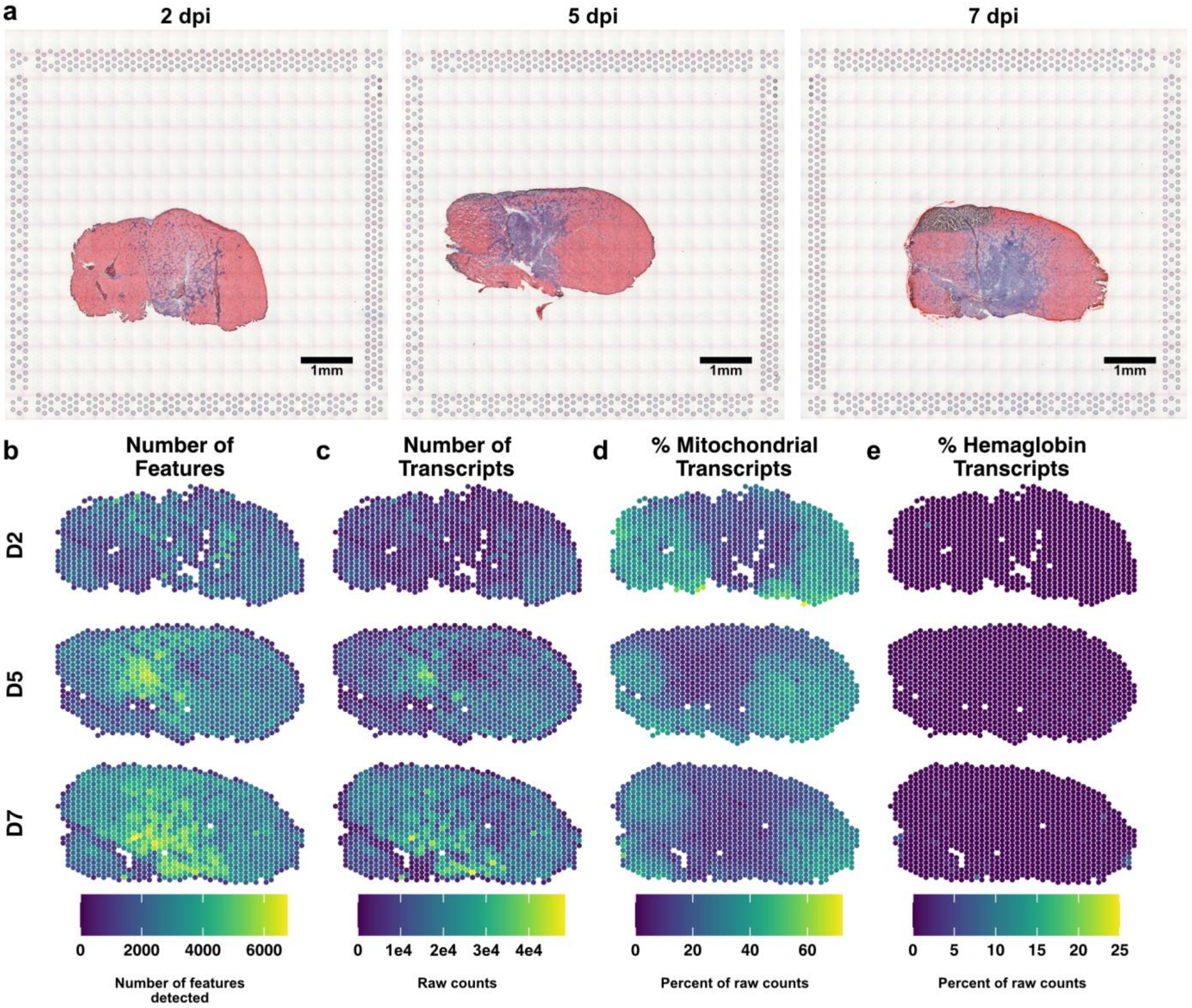

Figure S7. Quality metrics of Visium spatial RNA sequencing datasets. (a) Stitched H&E images of the samples used in Visium spatial RNA sequencing, including the perimeter of each frame. Each square is 6mm by 6 mm. **(b)** Number of features detected, **(c)** number of transcripts detected, **(d)** percent of unique transcripts mapping to mitochondrial genes, and **(e)** percent of unique transcripts mapping to hemoglobin genes, plotted for each spot. Samples shown are tibialis anterior muscles, collected two (D2), five (D5), or seven (D7) days post-injury (notexin).
