# Supplementary material for: Large-scale integration of single-cell transcriptomic data captures transitional progenitor states in mouse skeletal muscle regeneration
Source: Commun Biol. 2021 Nov 12;4:1280. doi: 10.1038/s42003-021-02810-x (PMC8589952; doi:10.1038/s42003-021-02810-x)
Supplement: Supplementary file 3 — Description of Additional Supplementary Files [file 42003_2021_2810_MOESM3_ESM.pdf]

Description of Additional Supplementary Files

File name: Supplementary Data 1
Description: Expanded table of metadata for all samples used in our analyses (related to **Table S1**).

File name: Supplementary Data 2
Description: Differential gene expression results for all cell types, including myogenic cell subsets; based on Harmony-integrated cell type annotations (output from Seurat::FindAllMarkers) (related to **Fig. S3**).

File name: Supplementary Data 3
Description: Differential gene expression results across myogenic cell subsets (output from Seurat::FindAllMarkers) (related to **Fig. 2**).

File name: Supplementary Data 4
Description: Differential gene expression results across endothelial cell subsets (output from Seurat::FindAllMarkers) (related to **Fig. S4**).

File name: Supplementary Data 5
Description: Differential gene expression results across FAP cell subsets (output from Seurat::FindAllMarkers) (related to **Fig. S4**).

File name: Supplementary Data 6
Description: Differential gene expression results across myeloid cell subsets (output from Seurat::FindAllMarkers) (related to **Fig. S4**).
