# Supplementary material for: Large-scale integration of single-cell transcriptomic data captures transitional progenitor states in mouse skeletal muscle regeneration
Source: Commun Biol. 2021 Nov 12;4:1280. doi: 10.1038/s42003-021-02810-x (PMC8589952; doi:10.1038/s42003-021-02810-x)
Supplement: Supplementary file 10 — Reporting Summary [file 42003_2021_2810_MOESM10_ESM.pdf]

nature research

Corresponding author(s): Cosgrove & De Vlaminck

Last updated by author(s): Sep 21, 2021

# Reporting Summary

Nature Research wishes to improve the reproducibility of the work that we publish. This form provides structure for consistency and transparency in reporting. For further information on Nature Research policies, see our Editorial Policies and the Editorial Policy Checklist.

## Statistics

For all statistical analyses, confirm that the following items are present in the figure legend, table legend, main text, or Methods section.

| n/a | Confirmed | |
|---|---|---|
| ☐ | ☒ | The exact sample size (*n*) for each experimental group/condition, given as a discrete number and unit of measurement |
| ☐ | ☒ | A statement on whether measurements were taken from distinct samples or whether the same sample was measured repeatedly |
| ☐ | ☒ | The statistical test(s) used AND whether they are one- or two-sided<br>*Only common tests should be described solely by name; describe more complex techniques in the Methods section.* |
| ☐ | ☒ | A description of all covariates tested |
| ☐ | ☒ | A description of any assumptions or corrections, such as tests of normality and adjustment for multiple comparisons |
| ☐ | ☒ | A full description of the statistical parameters including central tendency (e.g. means) or other basic estimates (e.g. regression coefficient) AND variation (e.g. standard deviation) or associated estimates of uncertainty (e.g. confidence intervals) |
| ☐ | ☒ | For null hypothesis testing, the test statistic (e.g. $F$, $t$, $r$) with confidence intervals, effect sizes, degrees of freedom and $P$ value noted<br>*Give P values as exact values whenever suitable.* |
| ☐ | ☒ | For Bayesian analysis, information on the choice of priors and Markov chain Monte Carlo settings |
| ☐ | ☒ | For hierarchical and complex designs, identification of the appropriate level for tests and full reporting of outcomes |
| ☐ | ☒ | Estimates of effect sizes (e.g. Cohen's $d$, Pearson's $r$), indicating how they were calculated |

*Our web collection on statistics for biologists contains articles on many of the points above.*

## Software and code

Policy information about availability of computer code

| | |
|---|---|
| Data collection | For all samples available via SRA, parallel-fastq-dump (github.com/rvalieris/parallel-fastq-dump) was used to download raw .fastq files. Samples which were only available as .bam files were converted to .fastq format using bamtofastq from 10x Genomics (github.com/10XGenomics/bamtofastq). |
| Data analysis | All code for processing and analysis of the scRNAseq and spatial RNA sequencing data, as well as supplemental data are available on Github (github.com/mckellardw/scMuscle). Raw reads were aligned to the mm10 reference using cellranger (v3.1.0). First, ambient RNA signal was removed using the default SoupX (v1.4.5) workflow (autoEstCounts and adjustCounts; github.com/constantAmateur/SoupX). Samples were then preprocessed using the standard Seurat (v3.2.1) workflow (NormalizeData, ScaleData, FindVariableFeatures, RunPCA, FindNeighbors, 356 FindClusters, and RunUMAP; github.com/satijalab/seurat). After preprocessing, DoubletFinder (v2.0) was used to identify putative doublets in each dataset, individually. After preprocessing and quality filtering, we merged the datasets and performed batch-correction with three tools, independently- Harmony (github.com/immunogenomics/harmony) (v1.0), Scanorama (github.com/brianhie/scanorama) (v1.3), and BBKNN (github.com/Teichlab/bbknn) (v1.3.12). We then used Seurat to process the integrated data. After initial integration, we removed the noisy cluster and re-integrated the data using each of the three batch-correction tools.<br><br>Cell types were determined for each integration method independently. For Harmony and Scanorama, dimensions accounting for 95% of the total variance were used to generate SNN graphs (Seurat::FindNeighbors). Louvain clustering was then performed on the output graphs (including the corrected graph output by BBKNN) using Seurat::FindClusters. A clustering resolution of 1.2 was used for Harmony (25 initial clusters), BBKNN (28 initial clusters), and Scanorama (38 initial clusters). Cell types were determined based on expression of canonical genes (Fig. S3). Clusters which had similar canonical marker gene expression patterns were merged.<br><br>Myogenic cells were subset based on the consensus cell types between all three integration methods. Harmony embedding values from the dimensions accounting for 95% of the total variance were used for further dimensional reduction with PHATE, using phateR (v1.0.4) (github.com/KrishnaswamyLab/phateR). |

Spot deconvolution was performed using the deconvolution module in BayesPrism (previously known as "Tumor microEnvironment Deconvolution", TED, v1.0; github.com/Danko-Lab/TED). First, myogenic cells were re-labeled, according to binning along the first PHATE dimension, as "Quiescent MuSCs" (bins 4-5), "Activated MuSCs" (bins 6-7), "Committed Myoblasts" (bins 8-10), and "Fusing Myoctes" (bins 11-18). Culture-associated muscle stem cells were ignored and myonuclei labels were retained as "Myonuclei (Type IIb)" and "Myonuclei Type IIx". Next, highly and differentially expressed genes across the 25 groups of cells were identified with differential gene expression analysis using Seurat (FindAllMarkers, using Wilcoxon Rank Sum Test; results in Sup. File 2). The resulting genes were filtered based on average log2-fold change (avg_logFC > 1) and the percentage of cells within the cluster which express each gene (pct.expressed > 0.5), yielding 1,069 genes. Mitochondrial and ribosomal protein genes were also removed from this list, in line with recommendations in the BayesPrism vignette. For each of the cell types, mean raw counts were calculated across the 1,069 genes to generate a gene expression profile for BayesPrism. Raw counts for each spot were then passed to the run.Ted function, using the "GEP" option for input.type and default parameters for the remaining inputs. Final Gibbs theta values were used as estimates for the fraction of transcripts from each spot that were derived from 397 each of the 25 cell types.

Ligand-receptor analysis and visualization was performed using CellChat v1.1.0 (github.com/sqjin/CellChat). The cell type labels used were derived from the Harmony integration results using all single-cell and single-nucleus data sources. Default values were used for the parameterization of each step.

For manuscripts utilizing custom algorithms or software that are central to the research but not yet described in published literature, software must be made available to editors and reviewers. We strongly encourage code deposition in a community repository (e.g. GitHub). See the Nature Research guidelines for submitting code & software for further information.

## Data

Policy information about availability of data

All manuscripts must include a data availability statement. This statement should provide the following information, where applicable:

- Accession codes, unique identifiers, or web links for publicly available datasets
- A list of figures that have associated raw data
- A description of any restrictions on data availability

Newly collected scRNAseq data for two samples from 7mo mice have been deposited in GEO under accession GSE159500. The data from 20mo mice will be released in GEO upon final publication. Spatial RNA sequencing data was deposited under GSE161318. A complete list of GEO accession numbers for the new and previously published scRNAseq data can be found in Table S1. SRR numbers for downloading each sample are compiled in Sup. File 1. The full integrated data with visualization tools is available at scmuscle.bme.cornell.edu.

# Field-specific reporting

Please select the one below that is the best fit for your research. If you are not sure, read the appropriate sections before making your selection.

☒ Life sciences ☐ Behavioural & social sciences ☐ Ecological, evolutionary & environmental sciences

For a reference copy of the document with all sections, see nature.com/documents/nr-reporting-summary-flat.pdf

# Life sciences study design

All studies must disclose on these points even when the disclosure is negative.

| | |
|---|---|
| Sample size | We generated 2-4 single-cell RNA sequencing replicates for each injury response time included within the study. Although no formal statistical analysis was used to determine the number of replicates, this extends beyond the standard number of replicates in the single-cell RNA sequencing field of n=1. We expanded the scope of our study by directly comparing this data to datasets previously generated in the lab (n=14) as well as many public datasets (n=76). Because of the costs and batch effects associated with each individual single-cell RNA sequencing sample, we argue that an additive approach in single-cell genomics is most effective for confirming the quality and fidelity of each individual sample. |
| Data exclusions | We applied thorough quality control to exclude single-cell transcriptomes which might be derived from dying cells (high percentage of reads aligning to mitochondrial genes), multiple cells, or empty droplets containing ambient RNA. These practices are widely used across the single-cell RNA sequencing field.<br>In collecting datasets for our analysis, we focused on mouse skeletal muscle samples collected using the 10x Genomics Chromium chemistry, to minimize batch effects, and excluded alternative chemistries. We also excluded samples from genetic knockout mice. |
| Replication | Within our compendium, we looked at samples collected from multiple ages, injury models, and genetic backgrounds to improve the robustness and repeatability of our findings. Within our computational pipeline, we solely used parameterization strategies which were well-defined and applicable to future studies. These factors both support the replicability of our work. |
| Randomization | Mice, from which injured hindlimb muscles were collected, were injured and collected so that multiple time points were collected at once (up to four samples in a single day), to reduce experimental bias. Mice were randomly selected for each injury time point. |
| Blinding | Blinding was not relevant for this study. |

# Reporting for specific materials, systems and methods

We require information from authors about some types of materials, experimental systems and methods used in many studies. Here, indicate whether each material, system or method listed is relevant to your study. If you are not sure if a list item applies to your research, read the appropriate section before selecting a response.

## Materials & experimental systems

| n/a | Involved in the study |
|---|---|
| ☒ | ☐ Antibodies |
| ☒ | ☐ Eukaryotic cell lines |
| ☒ | ☐ Palaeontology and archaeology |
| ☐ | ☒ Animals and other organisms |
| ☒ | ☐ Human research participants |
| ☒ | ☐ Clinical data |
| ☒ | ☐ Dual use research of concern |

## Methods

| n/a | Involved in the study |
|---|---|
| ☒ | ☐ ChIP-seq |
| ☒ | ☐ Flow cytometry |
| ☒ | ☐ MRI-based neuroimaging |

## Animals and other organisms

Policy information about studies involving animals; ARRIVE guidelines recommended for reporting animal research

| | |
|---|---|
| Laboratory animals | Adult C57BL/6J mice were obtained from Jackson Laboratories (#000664, Bar Harbor, ME) and were used at 4-7 months of age. Aged C57BL/6J mice were obtained from the National Institute of Aging (NIA) Rodent Aging Colony and were used at 20 months of age. For new scRNAseq experiments, female mice were used in each experiment. |
| Wild animals | This study did not involve wild animals. |
| Field-collected samples | This study did not involve samples collected from the field. |
| Ethics oversight | The Cornell University Institutional Animal Care and Use Committee (IACUC) approved all animal protocols, and experiments were performed in compliance with its institutional guidelines. |

Note that full information on the approval of the study protocol must also be provided in the manuscript.
